# I Know I Can, but Do I Have the Time? The Role of Teachers’ Self-Efficacy and Perceived Time Constraints in Implementing Cognitive-Activation Strategies in Science

**DOI:** 10.3389/fpsyg.2019.01697

**Published:** 2019-08-02

**Authors:** Nani Teig, Ronny Scherer, Trude Nilsen

**Affiliations:** Department of Teacher Education and School Research, Faculty of Educational Sciences, University of Oslo, Oslo, Norway

**Keywords:** cognitive activation, inquiry-based teaching, perceived time constraints, science education, science teaching, teacher self-efficacy, Trends in Mathematics and Science Study

## Abstract

Considerable research has demonstrated that teachers’ self-efficacy plays a major role in implementing instructional practices. Only few studies, however, have examined the interplay between how teachers’ self-efficacy and the challenges that lie outside their influence are related to their implementation of cognitive-activation strategies (CASs), especially in science classrooms. Using the Trends in Mathematics and Science Study 2015 data from science teachers in Grades 4, 5, 8, and 9, we explored the extent to which teachers’ self-efficacy in science teaching and the perceived time constraints explained variations in the enactment of general and inquiry-based CAS. Findings from the overall sample showed that highly self-efficacious teachers reported more frequent implementation of both general and inquiry-based CAS, whereas those who perceived strong time constraints reported a less frequent use of inquiry-based CAS. These relationships also existed across grade levels, except on the relations between perceived time constraint and inquiry-based CAS, which was only significant for the science teachers in Grade 9. We discuss these findings in light of variations in the core competencies of science curriculum, teachers’ competences, and the resources for science activities between primary and secondary education. We also point to the theoretical implications of this study for enhancing the conceptual understanding of generic and specific aspects of CAS and the practical implications for teacher education, professional development, and educational policy.

## Introduction

Challenging instruction has a key role for stimulating student learning. For this to happen, teachers need to provide students with cognitively activating learning opportunities that engage them in meaningful and higher-order thinking (Baumert et al., 2010). Cognitive-activation strategies (CASs) refer to challenging instructional approaches and learning tasks that stimulate students’ cognitive functioning and processing (Klieme et al., 2009; Lipowsky et al., 2009; Depaepe and König, 2018). CASs provide students with opportunities to foster an in-depth understanding of content through working on complex tasks, for example, by activating students’ prior knowledge, posing stimulating questions, and encouraging thoughtful discourse (for a review, see Seidel and Shavelson, 2007). Although prior research has shown that the enactment of CAS varies between teachers (Ryan et al., 2015; Künsting et al., 2016; Dorfner et al., 2017), few studies have examined the extent to which teacher beliefs can explain this variation, and even fewer studies have investigated it across grade levels, especially in science teaching. By focusing on two distinct aspects of teacher beliefs – self-efficacy and perceived time constraints – the current study aims to explain the variation in teachers’ implementation of CAS in science classrooms.

Research on science teaching almost exclusively focuses on supporting teachers to engage students in inquiry practices, such as formulating research questions, designing and conducting investigations, and analyzing and interpreting data (Blanchard et al., 2010; Minner et al., 2010) – activities that can be considered cognitively activating. As *subject-specific* practices, *inquiry-based CAS* are typically enacted for learning about science contents and the nature of science in more depth through first-hand experience in scientific investigations (Rönnebeck et al., 2016). Next to these practices, more *general CAS* go beyond inquiry-based teaching and comprise *generic* strategies aimed at fostering the development of conceptual knowledge through a range of practices, such as stimulating scientific discourse and questioning or linking new content to students’ prior knowledge (Klieme et al., 2009). Although general and specific aspects of CAS are both aimed at stimulating students’ cognitive engagement, the focus of their implementations in the classrooms can be different. Teachers who activate students cognitively would consider both types of CAS in their lessons – in fact, the competence to bring together general and domain-specific instructional approaches successfully is an important indicator of teacher quality (e.g., Shulman, 1986; Kulgemeyer and Riese, 2018).

Despite some evidence demonstrating the benefits of general CAS (e.g., Kane and Staiger, 2012; Mikeska et al., 2017) and inquiry-based CAS (e.g., Minner et al., 2010; Teig et al., 2018) for student learning, many teachers have not embraced the use of these strategies in their classrooms [see the international reports of Trends in Mathematics and Science Study (TIMSS) 2015 from Martin et al., 2016a and Programme for International Student Assessment (PISA) 2015 from OECD, 2016]. Teachers often feel a lack of confidence in enacting CAS in their practice (Murphy et al., 2007). These student-centered approaches are also demanding for teachers as they tend to be more time-consuming (Murphy et al., 2007; Powell-Moman and Brown-Schild, 2011; Smolleck and Mongan, 2011; Wang, 2011; Chen and Wei, 2015). Although previous research has indicated that teachers who feel confident in their teaching abilities are more likely to develop challenging lessons (Holzberger et al., 2014; Depaepe and König, 2018), it has not yet become clear how both teachers’ self-efficacy and the perceived time constraints are related to the implementation of CAS. Teachers who have low self-efficacy may perceive time constraints as a strong challenge, which hinders their application of CAS compared to those with high self-efficacy. However, these relationships may also vary between teachers in primary and lower secondary schools (henceforth referred to as secondary schools) due to the different instructional demands, curricula, and facilitating conditions.

Consequently, the present study focuses on investigating the extent to which teachers’ self-efficacy and perceived time constraints matter for the implementation of general and inquiry-based CAS in science across grade levels. The results could offer important insights into the role of teachers’ beliefs about their abilities and the facilitating conditions that support the use of engaging and cognitively challenging teaching strategies. These insights could have direct implications for teacher education and professional development as well as educational policy, as they reveal two potential aspects teachers may need to be supported with.

### General and Inquiry-Based Cognitive-Activation Strategies

Recently, researchers have emphasized the importance of investigating generic and subject-specific aspects of CAS for student learning and educational outcomes simultaneously (e.g., Mikeska et al., 2017; Charalambous and Praetorius, 2018). Despite this emphasis, CAS has been operationalized differently across studies, and the distinction between generic and subject-specific aspects still require conceptual and empirical support (Schlesinger and Jentsch, 2016). For instance, focusing on mathematics instruction, some researchers emphasized aspects such as activating prior knowledge and working on challenging tasks as key elements of CAS (Klieme et al., 2009; Baumert et al., 2010), while others focused on engaging students in thoughtful discourse or using instructional scaffolding (e.g., Kane and Staiger, 2012; Pianta et al., 2012; Schoenfeld, 2013). Although these aspects are situated within mathematics instruction, they are also relevant in other subjects including science (Schlesinger and Jentsch, 2016). As such, despite their dependence on subject-specific knowledge and contents, they may therefore be considered *general CAS*.

At the same time, as a subject-specific instruction, inquiry-based teaching that represents scientific practices lies at the heart of science teaching and has long been advocated by science education communities (e.g., American Association for the Advancement of Science, 1994; Rocard et al., 2007). Learning science through investigation places a strong emphasis on students’ active learning and their responsibility for constructing their own knowledge (Rönnebeck et al., 2016; Kaiser et al., 2018). Such activities are particularly unique to science teaching, as they are not typically used in other subject domains. Inquiry practice activates cognitive processing and fosters students’ reasoning and thinking skills. In the current study, such instructional approaches are considered to be subject-specific CAS and referred to as *inquiry-based CAS*. By applying the framework of inquiry-based learning from Pedaste et al. (2015) and Rönnebeck et al. (2016), inquiry is simplified as the practice in which students design or plan experiments, conduct experiments to collect evidence, interpret the evidence from the experiments, use the evidence to justify conclusions, and communicate the results of the experiments (Supplementary Figure S1).

Although a conceptual overlap seems to exist between general and subject-specific CAS, knowledge about their commonalities, differences, and the extent to which they are related is limited (Schlesinger and Jentsch, 2016). This is surprising because the conceptualization of teaching practices as both domain-general and domain-specific directly informs both teacher education and professional development (e.g., Loewenberg Ball and Forzani, 2009; Barrera-Pedemonte, 2016).

### Self-Efficacy in Science Teaching

Self-efficacy is an important teacher characteristic that is closely connected to instructional quality and successful student learning (Holzberger et al., 2014; Schiefele and Schaffner, 2015). Teacher self-efficacy refers to the teachers’ beliefs in their abilities to successfully enact critical instructional tasks in a particular context (Tschannen-Moran et al., 1998). According to this definition, teacher self-efficacy is a result of the interaction between the evaluation of factors that contribute to teaching difficulties and individual perceptions of teaching abilities. Self-efficacy beliefs are considered multifaceted constructs that differ across contexts and that comprises multiple sources (Bandura, 1977; Tschannen-Moran et al., 1998). In the context of our study, we focus on teacher self-efficacy in science teaching, that is, teachers’ judgments of their capabilities to implement instructional strategies in science that can influence student learning positively (Riggs and Enochs, 1990; Palmer, 2006; Cakiroglu et al., 2012).

A mounting body of evidence demonstrates the relevance of teachers’ self-efficacy to their instructional behaviors (see review by Mansour, 2009; Zee and Koomen, 2016). In particular, teachers with a high sense of self-efficacy are more likely to develop challenging lessons, provide more autonomy for student learning (Tschannen-Moran et al., 1998; Sandholtz and Ringstaff, 2014), experiment with new instructional strategies, and try different teaching materials compared to teachers with lower self-efficacy (McKinnon and Lamberts, 2014). They also show greater commitment to improving their teaching and are more persistent in working with challenging students (Tschannen-Moran et al., 1998; Sandholtz and Ringstaff, 2014). Recent studies have also revealed positive and significant relationships between teacher self-efficacy and all three dimensions of instructional quality: classroom management, supportive climate, and cognitive activation (e.g., Künsting et al., 2016; Depaepe and König, 2018). These relations suggest that highly self-efficacious teachers manage classrooms well, establish a supportive classroom climate, and activate students cognitively. Overall, these relations suggest a link between teachers’ self-beliefs and their performance during instruction (see also Vieluf et al., 2013; Zee et al., 2016; Daniels et al., 2017). However, teachers’ use of CAS showed the weakest link to teacher self-efficacy among the instructional quality dimensions. Künsting et al. (2016) explained that this finding resulted from a lack of alignment between the self-efficacy and instructional quality measures. More specifically, they argued that the scale used to capture teacher self-efficacy was somewhat less relevant for CAS compared to other dimensions of instructional quality. In the current study, we consequently use a teacher self-efficacy measure that focuses on the CAS aspect of science instruction rather than general science instruction – the latter would also include other aspects, such as teacher support and classroom management. This alignment could enhance a conceptual relevance between the self-efficacy measure and the measure of CAS as teaching practices.

Prior research also indicated differences in teachers’ self-efficacy across academic levels (e.g., Martin et al., 2012; OECD, 2014; Ryan et al., 2015). According to TIMSS 2015, primary school teachers seemed to have lower self-efficacy in science teaching compared to teachers in secondary schools (Martin et al., 2012). On average, across the TIMSS participating countries, only 59% of fourth-grade students had teachers who were confident in teaching science compared to 73% of eighth-grade students. Most teachers reported low self-efficacy in providing challenging tasks for capable students; the fourth-grade teachers felt particularly the least confident in explaining science concepts or principles by conducting science experiments, whereas the eighth-grade teachers were least confident in adapting their teaching to engage student interests. Other studies, such as the ones conducted by Holroyd and Harlen (1996) and Murphy et al. (2007), also highlighted the continuous trend for the lack of primary school teachers’ confidence in science teaching over the past decades. Previous research identified teachers’ *mastery experience* as a critical source of their self-efficacy, especially for in-service primary science teachers (e.g., Palmer, 2006, 2011). Teachers’ perceived success in cognitive mastery (understanding science or pedagogical concepts) and enactive mastery (performing science teaching) was important aspects that contribute to fostering teacher self-efficacy from both short- and long-term perspectives (Palmer, 2011; McKinnon and Lamberts, 2014; Menon and Sadler, 2016). Since primary school teachers seem to have few opportunities for enhancing their mastery experience (Palmer, 2011; Martin et al., 2012), they might feel less confident to engage their student with cognitively challenging science lessons, compared to teachers in secondary schools.

Although teacher self-efficacy has been shown to predict the implementation of CAS (e.g., Holzberger et al., 2014; Künsting et al., 2016), the extent to which teacher self-efficacy is related to both general and subject-specific CAS, particularly in science teaching, is largely unknown. In addition, we know little about the potential differences in these relationships as a function of grade level between primary and secondary schools. A more comprehensive understanding of how these relations may be similar or differ across academic levels would be important in developing relevant curricula and interventions in teacher training and professional development to better support teachers in enacting CAS. Knowledge about possible differences in the abovementioned relations may also help teacher educators to promote the development of teachers’ adaptive teaching expertise – an expertise that helps them adjust instructional practices to students’ backgrounds, competences, and needs (Soslau, 2012).

### Perceived Time Constraints

Time plays an important role in understanding teachers’ pedagogical decisions. Teachers who perceive less pressure at work are more likely to be self-determined toward teaching and implement student-directed instruction that gives students greater freedom to learn (Pelletier et al., 2002). Given the complexity of cognitively activating instruction, the time allocated for its implementation is critical. Empirical studies have identified teachers’ perceived time constraints as obstacles that hindered their decision to enact CAS (e.g., Newman et al., 2004; Murphy et al., 2007; Wang, 2011; Chichekian and Shore, 2016). A recent study by Hofer et al. (2018), which was designed to enhance students’ conceptual understanding of physics with the use of CAS, highlighted the necessity of devoting adequate time to actively involve students in the process of knowledge construction. Drawing from Ajzen’s Theory of Planned Behavior – a theory that describes the links between a person’s beliefs about him- or herself, the external conditions that may facilitate certain behavior, the usefulness of this behavior, and the ease of this behavior (Ajzen, 1991) – we argue that perceived time constraints represent facilitating conditions that may directly or indirectly determine teachers’ intentions to implement CAS and their actual use of CAS in science classrooms.

Teachers’ perceptions of the time constraints might relate differently to general and inquiry-based CAS. Depending on the content being taught, learning activities that include general CAS – such as dialogic classroom interaction – might require less time compared to inquiry-based CAS, which entails several learning phases that build on each other in a systematic way. Along this line, we explore whether and to what extent the perceived time constraints are related to the implementation of general and inquiry-based CAS.

### The Present Study

Taking advantage of a large, high-quality dataset from TIMSS 2015, we investigate the interplay between teacher self-efficacy in science teaching and the perceived teaching challenges related to time constraints as variables that may explain variation in the implementation of CAS. First, due to the complexity of CAS, distinguishing between the generic and specific aspects of CAS and providing empirical evidence on the relevance of this distinction were critical steps in the present study to develop a more comprehensive understanding of CAS. Attending to the generic as well as subject-specific CAS is crucial to better understand the complex process of teachers’ instructional decision-making that results in more effective science teaching (Charalambous and Kyriakides, 2017; Mikeska et al., 2017).

Second, we examine the relationships of teachers’ perceptions of self-efficacy and time constraints with their use of general and inquiry-based CAS in science. Although recent evidence has demonstrated the reverse effects between teachers’ self-efficacy and classroom practices (e.g., Holzberger et al., 2013), and it seems plausible to suggest that the association between teachers’ perceived time constraints and their instructions might be reciprocal, it is not the scope of this study to determine the direction of causality. The present study focuses on teachers’ perceptions of their self-efficacy and the time constraints in teaching – the latter represents the challenges to teaching that lie outside of teachers’ influence – to explain the variation in the enactment of general and inquiry-based CAS in science classrooms. These different beliefs could play a significant role on the amount of effort that goes into teaching and on the pedagogical choice to implement CAS.

Third, given the possible differences between primary and secondary schools, we compare the relations of teacher beliefs and CAS across Grades 4, 5, 8, and 9. These relations may further vary across countries (Blömeke et al., 2016), and this is one of the first studies to examine such variations in a Norwegian context. The Norwegian compulsory education system consists of primary school (Grades 1–7) and secondary school (Grades 8–10). A transition also occurs between lower primary school (Grades 1–4) and upper primary school (Grades 5–7), which covers a shift toward more complex learning goals, specialized textbooks, and, in some places, a change of school. An important difference also exists between Grades 8 and 9. In Norway, students start to receive grades in secondary schools – hence, teachers’ instructional practice in Grade 8 emphasizes easing the transition process from primary to secondary school, gradually introducing performance assessments. Moreover, investigating the differences in relationships between teachers’ beliefs and their use of CAS across grades is part of a robustness check of the findings that accounts for the various transitions associated with the Norwegian school context.

The effectiveness of CAS implementation depends on several components, including the teacher, the students within the classroom, the necessary teaching resources, and their interactions. Going beyond the existing research, this study provides insights into the generic and specific aspects of CAS, as well as the roles of teachers’ self-beliefs and the perceived time constraints for engaging students in CAS across grade levels.

## Materials and Methods

### Sample and Procedure

The data were derived from the TIMSS study, an international large-scale survey that compares trends in mathematics and science performance in participating countries every fourth year (Martin et al., 2016a). TIMSS uses a two-stage stratified cluster design in choosing participants within a country – first, schools are sampled and then intact classrooms of students are selected randomly within the participating schools (see Martin et al., 2016b for further details). Additionally, TIMSS collects data from teachers, school leaders, students, and parents, focusing on contextual variables related to student learning.

The current study utilized science teacher data from the Norwegian TIMSS conducted in 2015. In this cycle, Norway changed the target population of students from Grades 4 and 8 to Grades 5 and 9 to improve the comparability to other Nordic countries (Bergem et al., 2016a; Kavli, 2018). Specifically, whereas Norwegian children start school at the age of 6 years, Swedish, Danish, and Finish children start school at the age of 7 years. As a consequence, TIMSS 2015 included not only the samples of fourth and eighth graders (i.e., benchmark samples), but also the samples of fifth and ninth graders. Using the Norwegian TIMSS 2015 data allowed not only for sampling across grade levels in primary and secondary schools but also for testing the robustness of the findings across grade levels.

The sample consisted of *N* = 804 science teachers (62.9% female; 74.9% under the age of 50 years; teaching experience: *M* = 13.1, *SD* = 10.1 years). Detailed teacher characteristics are shown in Table 1. Note that teachers implement an integrated science curriculum in primary and lower secondary schools.

**TABLE 1:** Percentages of teacher characteristics across grade levels.

**Variables**	**Grade**			
	**4**	**5**	**8**	**9**
*N* teachers/classrooms	193	187	213	211
Gender				
Male	21.8	30.9	45.4	47.4
Female	78.2	69.1	54.6	52.6
Years of teaching experience				
<10 years	38.2	41.0	45.9	44.8
10–19 years	33.6	34.9	35.7	34.6
20–30 years	18.2	12.7	10.7	8.7
≥30 years	10.0	11.4	7.7	11.9
Level of formal education				
Upper secondary	0.6	0.6		
Post-secondary	0.7	1.2		
Short-cycle tertiary	9.5	6.0	2.6	5.2
Bachelor or equivalent	82.7	84.3	70.9	68.0
Master or equivalent	6.3	7.8	26.0	25.8
Doctor or equivalent			0.50	1.00
Major area of education^a^				
Primary education	83.4	86.7		
Secondary education	9.5	8.6		
Primary/secondary education:				
Specialization in mathematics	27.7	31.1		
Specialization in science	28.3	37.0		
Mathematics	26.2	34.1	58.7	53.9
Science:	26.6	36.6		
Biology			36.9	31.1
Physics			12.8	14.0
Chemistry			30.9	21.8
Earth science			5.7	6.7
General education			63.1	62.2
Mathematics education			15.5	17.2
Science education			25.8	25.1

*^*a*^ Teachers are allowed to choose more than one option for their major area of education.*

### Measures

#### CAS in Science Teaching

In TIMSS 2015, teachers were asked about their perceptions of the frequency of various activities in their classrooms. They indicated the frequency with which 22 teaching and learning activities occurred in their science lessons using a four-point Likert scale (from 0 = *never* to 3 = *every or almost every lesson*). Of these 22 items, we chose 11 items that were related to CAS in science teaching: six items representing general CAS (e.g., asking students to complete challenging exercises that require them to go beyond the instruction) and five items representing inquiry-based CAS (e.g., designing or planning experiments or investigations).

#### Self-Efficacy in Science Teaching

Teachers were asked to rate their confidence in performing 10 science teaching tasks related to CAS on a four-point Likert scale (from 0 = *low* to 3 = *very high*). The items referred to the degree to which they believed they could do these tasks (e.g., developing students’ higher-order thinking skills).

#### Perceived Time Constraints

Teachers were asked to indicate their level of agreement with six different statements that reflect teaching challenges related to time constraints (e.g., I need more time to prepare for class) using a four-point Likert scale that ranged from 0 (*disagree a lot*) to 3 (*agree a lot*).

For further details on item wordings and labels as well as descriptive statistics of all the measures from the total sample and each grade sample, please refer to Supplementary Table S1. The complete teacher questionnaires and detailed information about the scaling and validation process of the scales across countries and grade levels are available at the TIMSS 2015 website^1^. The items for general and inquiry-based CAS can be found at sections G14 and S3 (Grades 4 and 5) and sections 14 and 18 (Grades 8 and 9), the items for teacher self-efficacy in science teaching are available at section S2 (Grades 4 and 5) and section 17 (Grades 8 and 9), whereas the items for teachers’ perception of time constraints are presented in section G11 (Grades 4 and 5) and section 11 (Grades 8 and 9).

### Data Analysis

The teacher data were imported from TIMSS international database^2^, prepared using the IDB Analyzer Version 4.0, and further analyzed with the statistical software *Mplus* 7.4 (Muthén and Muthén, 1998-2018). The rate of missing data ranged from 9.7% to 15.8% at the level of item responses, and the full information maximum-likelihood estimation was used to handle the missingness (Enders, 2010). To correct standard errors in the presence of missing data and possible deviations from normality, the robust maximum-likelihood estimator was used. All model comparisons involving chi-square statistics are therefore corrected according to Satorra and Bentler (2010) procedure. Furthermore, we used the TYPE = COMPLEX option to take into account the nesting of the teacher data in schools (Muthén and Muthén, 1998-2018).

The data analysis focused on (a) establishing measurement models to represent general and specific CAS in science teaching, teacher self-efficacy, and perceived time constraints; (b) examining the relations among these constructs for the full sample; and (c) examining the relations among these constructs across grade levels. To accomplish (a), we performed explanatory factor analysis (EFA) and confirmatory factor analysis (CFA). For each construct, we employed EFA to examine the items that were related to the construct and inspected their underlying dimensions. Next, we conducted CFA to verify the underlying dimensions of the construct and, ultimately, obtain information about the model fit to the data. For each construct, we specified a measurement model that reflected our theoretical assumptions on the constructs, first for the total sample and then for the samples of students in Grades 4, 5, 8, and 9. The second step was taken to ensure that each measurement model formed an appropriate baseline and construct representation in each grade. We evaluated the model fit using common goodness-of-fit indices and their guidelines for an acceptable fit [root mean square error of approximation (RMSEA) ≤ 0.08, comparative fit index (CFI) ≥ 0.95, Tucker-Lewis index (TLI) ≥ 0.95, and standardized root mean square residual (SRMR) ≤ 0.10; Marsh et al., 2005]. Notice that these guidelines do not represent “golden rules” as they depend on the specific features of the measurement models, such as the number of factors, the type of factor structure, and the sample size (Marsh et al., 2004).

Based on the measurement models established in the previous steps, we performed structural equation modeling to examine the relations among the latent variables, both for the full sample and for the sample across grades. Further, we controlled for teachers’ gender, years of teaching experience, and educational level as these variables have shown to be significantly related to teachers’ self-efficacy (e.g., Klassen and Chiu, 2010; Tuchman and Isaacs, 2011) by adding them as covariates of teachers’ self-efficacy construct. For the full sample, we began with specifying the relations between teacher self-efficacy and CAS in science teaching and then added perceived time constraints to the structural model. Prior to investigating the differential relations of the constructs between grades, it was essential to assess the invariance of the measurement models across grade levels by applying multi-group CFA to accomplish this (Sass and Schmitt, 2013; Greiff and Scherer, 2018). We started with the model that assumed the same factor structure across grade levels, yet without equality constraints of the model parameters (configural invariance) and then constrained the factor loadings (metric invariance) to be equal across grades. If at least metric invariance was obtained (i.e., teachers interpreted the constructs similarly across grade levels), we tested whether the relations between the constructs were equal across grades (structural or relational invariance). For comparing the freely estimated with the constrained models in the measurement and structural invariance testing, we used the Satorra–Bentler corrected chi-square difference test (SB-χ^2^, Satorra and Bentler, 2010) and/or the differences in fit indices (ΔCFI ≥ −0.01, ΔRMSEA ≥ 0.014, and ΔSRMR ≥ 0.015 as evidence of non-invariance; Chen, 2007). Under the condition of unequal structural relations across grades, we further performed the Wald test of parameter constraints to test the specific differences in the relations between pairs of grade levels (Brown, 2015).

## Results

In the following section, we first present the results of the preliminary analyses that were aimed at establishing appropriate measurement models for each construct. Next, we present the overall findings on the relations among the constructs for the total sample and more detailed results on how these relations may vary across grade levels. These findings are supplemented by the results of measurement and structural invariance testing.

### Preliminary Analyses

#### Descriptive Statistics and Correlations

In Table 2, we summarized the score means, standard deviations, and correlations among the constructs for the full sample and for each grade level. In general, the means were relatively high, and the magnitude of correlations was low to moderate. With respect to the full sample, the correlations between teachers’ self-efficacy and their implementations of general and inquiry-based CAS were positive, yet negative between the perceived time constraints and inquiry-based CAS. We found similar relations for each grade level, except for Grade 8, in which the negative correlation between the perceived time constraints and inquiry-based CAS was not significant. Hence, high self-efficacious teachers reported a more frequent implementation of CAS, whereas teachers who perceived stronger time constraints used less CAS in their instructions. In addition, the correlations between general and inquiry-based CAS were low (*r*s = 0.32–0.52), pointing to the distinction between these two aspects of CAS.

**TABLE 2:** Mean scores, standard deviations, and correlations matrices for the constructs.

**Constructs**	M	SD	**1.**	**2.**	**3.**	**4.**
**Full sample (*N =* 804 teachers)**						
1. Self-efficacy	1.83	0.69	1.00	−	−	–
2. Time constraints	1.99	0.85	−0.11^*^	1.00	−	–
3. General CAS	1.86	0.74	0.37^∗∗∗^	–0.05	1.00	–
4. Inquiry-based CAS	1.16	0.53	0.39^∗∗∗^	–0.15^∗∗^	0.39^∗∗∗^	1.00
**Grade 4 (*N =* 193 teachers)**						
1. Self-efficacy	1.74	0.67	1.00	−	−	–
2. Time constraints	2.01	0.84	–0.13	1.00	−	–
3. General CAS	1.86	0.74	0.24^∗∗^	–0.13	1.00	–
4. Inquiry-based CAS	0.99	0.46	0.23^∗∗^	–0.18^∗∗^	0.32^∗∗^	1.00
**Grade 5 (*N* = 187 teachers)**						
1. Self-efficacy	1.75	0.72	1.00	−	−	–
2. Time constraints	1.96	0.88	–0.16	1.00	−	–
3. General CAS	1.89	0.75	0.30^∗∗^	0.15	1.00	–
4. Inquiry-based CAS	1.16	0.51	0.57^∗∗∗^	0.01^*^	0.39^∗∗∗^	1.00
**Grade 8 (*N =* 213 teachers)**						
1. Self-efficacy	1.91	0.68	1.00	−	−	–
2. Time constraints	2.02	0.82	0.01	1.00	−	–
3. General CAS	1.83	0.73	0.54^∗∗∗^	–0.10	1.00	–
4. Inquiry-based CAS	1.22	0.53	0.47^∗∗∗^	–0.08	0.48^∗∗∗^	1.00
**Grade 9 (*N =* 211 teachers)**						
1. Self-efficacy	1.91	0.67	1.00	−	−	–
2. Time constraints	1.97	0.86	–0.17	1.00	−	–
3. General CAS	1.82	0.72	0.48^∗∗∗^	–0.13	1.00	–
4. Inquiry-based CAS	1.24	0.54	0.40^∗∗∗^	–0.17^∗∗^	0.52^∗∗∗^	1.00

^*^p < 0.05, ^∗∗^*p* < 0.01, ^∗∗∗^*p* < 0.001.

#### Measurement Models

As noted earlier, the conceptualization of CAS as a key dimension of teaching quality varies across studies. CAS can contain both generic features of instruction that are similar across subjects and domain-specific teaching strategies (Schlesinger and Jentsch, 2016). To test this assumption, we applied EFA with a geomin rotation to the 11 CAS items. The list of eigenvalues favored a two-factor model of CAS and provided an interpretable pattern of factor loadings. Specifically, the first factor was indicated by the items representing general CAS, whereas the second factor was indicated by the inquiry-related CAS items (Table 3). The resultant factor correlation was moderate with *r* = 0.38. The screeplot of eigenvalues along with the reference values of the Empirical Kaiser Criterion (EKC; Braeken and van Assen, 2017) is presented in Supplementary Figure S2.

**TABLE 3:** The results of exploratory factor analysis for CAS.

**Item label**	**Item wording**	**Eigenvalue**	**Factor loadings**	
			General CAS	Inquiry-based CAS
Live	Relate the lesson to students’ daily lives	3.833	0.417	
Chal	Ask students to complete challenging exercises that require them to go beyond the instruction	1.876	0.476	
Disc	Encourage classroom discussions among students	0.903	0.641	
Link	Link new content to students’ prior knowledge	0.834	0.539	
Prob	Ask students to decide their own problem-solving procedures	0.702	0.648	
Idea	Encourage students to express their ideas in class	0.614	0.634	
Expl	Design or plan experiments or investigations	0.585		0.617
Expr	Conduct experiments or investigations	0.521		0.728
Data	Interpret data from experiments or investigations	0.497		0.808
Com	Present data from experiments or investigations	0.373		0.832
Con	Use evidence from experiments or investigations to support conclusions	0.260		0.563

*Factor loadings less than ±0.20 were excluded.*

**FIGURE 1:**
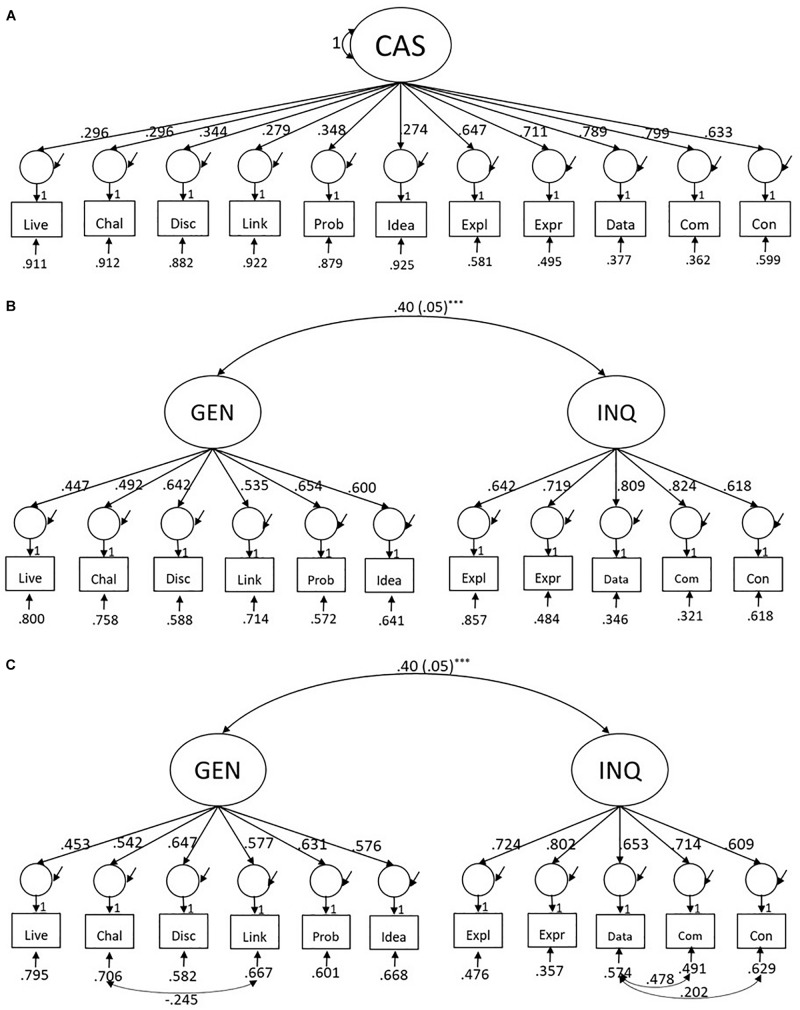
Comparison between **(A)** the one-factor model of CAS, **(B)** the two-factor model of CAS, and **(C)** the two-factor model of CAS with correlated factors for the total sample. Latent variables: CAS, cognitive-activation strategies; GEN, general CAS; INQ, inquiry-based CAS. Please refer to Supplementary Table S1 for further details of the item labels and wordings as well as the descriptive statistics of these measures.

Using CFA, we further verified the EFA results by evaluating whether a two-factor model represented the data better than a one-factor model of CAS – the former contained two correlated factors of CAS (i.e., general and inquiry-based CAS). We conducted this model comparison both for the full sample and for each grade level. For the total sample, the scale reliability of the one-factor CAS model was acceptable (ω = 0.79), yet the model indicated a poor fit, SB-χ^2^(44) = 652.8, *p* < 0.001, RMSEA = 0.141, CFI = 0.678, TLI = 0.597, SRMR = 0.111 (Figure 1A). In contrast, the two-factor CAS model resulted in a reasonable fit [SB-χ^2^(43) = 197.3, *p* < 0.001, RMSEA = 0.072, CFI = 0.918, TLI = 0.895, SRMR = 0.043] with acceptable scale reliabilities of ω = 0.76 and ω = 0.78 and sufficiently high factor loadings that ranged from 0.45 to 0.65 and 0.62 to 0.82 for general and inquiry-based CAS, respectively (Figure 1B). The factor correlation between general and inquiry-based CAS was low, ρ = 0.40, *p <* 0.001. The chi-square difference test that compared the two competing models (Figures 1A,B) suggested a significantly better fit for the two-factor CAS model, ΔSB-χ^2^(1, *N* = 692) = 499.0, *p* < 0.001. Finally, due to the conceptual and methodological reasons (i.e., some learning activities are intertwined, such as interpreting and presenting data from experiments, and the suggestions from the modification indices to improve the model fit), we added three correlations among item residuals that led to the refined two-factor model of CAS presented in Figure 1C. This final model of CAS indicated an excellent fit [SB-χ^2^(40) = 85.2, *p* < 0.001, RMSEA = 0.040, CFI = 0.976, TLI = 0.967, SRMR = 0.036] and outperformed the two-factor model without residual correlations, ΔSB-χ^2^(3, *N* = 692) = 129.3, *p* < 0.001. We therefore accepted the two-factor model with residual correlations as the measurement model of CAS for the total sample.

To test whether the measurement model of CAS holds for the different grade levels, we conducted the same analyses for the data from each grade and found that the results pointed into the same direction as those obtained from the total sample. In Table 4, we provide detailed results of the fit indices and difference tests for model comparisons. In general, the chi-square difference tests suggested that the two-factor model of CAS with residual correlations had a better fit than the one-factor model for each grade level. For these reasons, we used the two-factor model with residual correlations as the baseline measurement model of CAS for further analyses. This model formed the basis for examining how different aspects of CAS were related to teachers’ self-efficacy and the perceived time constraints in science teaching.

**TABLE 4:** Model fit statistics for the measurement models of CAS.

**Model**	**LL**	**SCF**	**Npar**	**RMSEA**	**CFI**	**TLI**	**SRMR**	**Model comparisons^a^ ΔSB-χ^2^ (Δ*df*)**	
								**M1 vs. M2**	**M2 vs. M3**
**Full sample**									
M1: One-factor model	−6421.3	1.39	33	0.141	0.678	0.597	0.111		
M2: Two-factor model	−6171.8	1.34	34	0.072	0.918	0.895	0.043	499.0 (1)^∗∗∗^	
M3: Two-factor model with residuals	−6107.2	1.35	37	0.040	0.976	0.967	0.036		129.3 (3)^∗∗∗^
**Grade 4**									
M1: One-factor model	−1442.6	1.43	33	0.149	0.632	0.539	0.124		
M2: Two-factor model	−1380.9	1.46	34	0.085	0.884	0.852	0.064	123.4 (1)^∗∗∗^	
M3: Two-factor model with residuals	−1359.4	1.49	37	0.050	0.963	0.949	0.058		43.0 (3)^∗∗∗^
**Grade 5**									
M1: One-factor model	−1412.3	1.29	33	0.126	0.736	0.670	0.120		
M2: Two-factor model	−1355.6	1.30	34	0.052	0.956	0.944	0.058	113.3 (1)^∗∗∗^	
M3: Two-factor model with residuals	−1344.9	1.29	37	0.024	0.991	0.987	0.056		21.4 (3)^∗∗∗^
**Grade 8**									
M1: One-factor model	−1733.5	1.21	33	0.136	0.703	0.629	0.100		
M2: Two-factor model	−1677.5	1.17	34	0.071	0.922	0.900	0.055	112.0 (1)^∗∗∗^	
M3: Two-factor model with residuals	−1665.5	1.17	37	0.053	0.959	0.944	0.052		24.1 (3)^∗∗∗^
**Grade 9**									
M1: One-factor model	−1699.9	1.29	33	0.140	0.710	0.638	0.101		
M2: Two-factor model	−1643.8	1.25	34	0.080	0.907	0.882	0.056	112.3 (1)^∗∗∗^	
M3: Two-factor model with residuals	−1619.9	1.22	37	0.039	0.980	0.972	0.043		47.7 (3)^∗∗∗^

LL, log-likelihood value; SCF, scaling correction factor; Npar, number of parameters; RMSEA, root mean square error of approximation; CFI, comparative fit index; TLI, Tucker–Lewis index; SRMR, standardized root mean square residual; SB-χ^2^, Satorra–Bentler corrected chi-square statistic; *df*, degrees of freedom. ^*a*^ The difference test for model comparisons is based on Satorra–Bentler chi-square difference test, which produced corrected Δχ^2^ statistics when MLR is used as the maximum likelihood estimator. ^*^p < 0.05, ^∗∗^*p* < 0.01, ^∗∗∗^*p* < 0.001.

Using the same steps of analysis, we investigated the measurement models of teacher self-efficacy and perceived time constraint, both for the total sample and for the grade-specific samples (Supplementary Figure S3). For teacher self-efficacy, a one-factor CFA model showed an acceptable fit to the data of the total sample [SB-χ^2^(33) = 155.7, *p* < 0.001, RMSEA = 0.074, CFI = 0.960, TLI = 0.945, SRMR = 0.031] and a satisfactory scale reliability of ω = 0.92 with high factor loadings that ranged from 0.71 to 0.77. For teachers’ perceived time constraints, the one-factor model of CFA resulted in a good model fit [SB-χ^2^(8) = 24.6, *p* < 0.001, RMSEA = 0.054, CFI = 0.979, TLI = 0.960, SRMR = 0.026], the scale reliability was acceptable (ω = 0.82), and the factor loadings ranged from 0.53 to 0.79. These models could be retained for the grade-specific samples (Supplementary Table S2). Along with the two-factor model representing CAS, the one-factor models representing teacher self-efficacy and the perceived time constraints formed the basis for all subsequent analyses.

### Relations Among Latent Variables for the Full Sample

**FIGURE 2:**
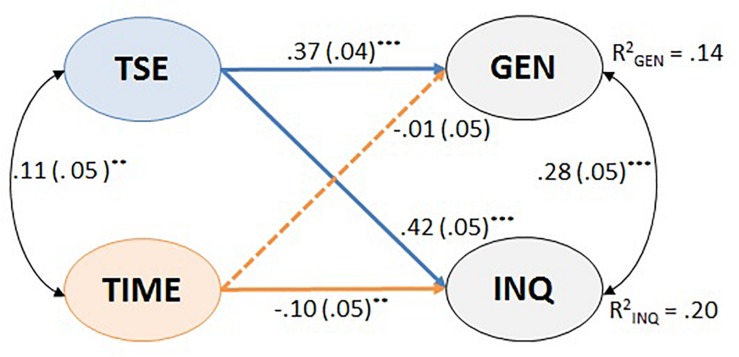
Structural equation model representing the relations among the latent variables for the total samples. Latent variables: TSE, teacher self-efficacy in science teaching; TIME, teachers’ perception of time constraints; GEN, general CAS; INQ, inquiry-based CAS. ^*^p < 0.05, ^∗∗^*p* < 0.01, ^∗∗∗^*p* < 0.001.

We combined the measurement models of CAS, teacher self-efficacy in science teaching, and the perceived time constraints to examine their structural relations. The combined model exhibited an acceptable fit to the data, SB-χ^2^(312) = 611.2, *p <* 0.001, RMSEA = 0.036, CFI = 0.955, TLI = 0.950, SRMR = 0.038. As shown in Figure 2, the model explained 14% of the variance in general CAS and 20% of the variance in inquiry-based CAS. Teachers’ self-efficacy was positively related to both general and inquiry-based CAS. Likewise, their perceptions about time constraints were negatively related to inquiry-based CAS, although they were not significantly related to general CAS. Furthermore, the associations remained after controlling for teachers’ gender, their years of teaching experience, and their educational level.

### Relations Among Latent Variables Across Grades

#### Measurement Invariance Testing

We further investigated whether the measurement models were invariant across grade levels. This analytical step forms the prerequisite for comparing relations among variables across groups (e.g., Brown, 2015). As presented in Table 5, SB-χ^2^ tests were insignificant for all the constructs. Moreover, the results showed that all constructs exhibited values below the suggested criteria for all changes in fit indices (Chen, 2007), except for the construct of perceived time constraints that had ΔCFI of −0.025. Nevertheless, Chen (2007) has also suggested that these criteria should be implemented with caution as measurement invariance testing is a very complex issue that could be affected by various factors, such as sample size, model complexity, and pattern of invariance. The suggested criteria for change in fit indices were investigated under limited conditions, and a number of factors could influence the magnitude of changes. For instance, the present study took into account the fact that teachers were clustered within schools, which was not considered in Chen’s simulation study (2007). From this perspective, the analyses confirmed metric invariance across grades; constraining factor loadings of the corresponding indicators to be equal across grades led to an insignificant decrease in the model fit indices. Attaining metric invariance for all the constructs under investigation is critical for making valid comparison as these results implied that these constructs have the same conceptual interpretation for the teachers across grades. Since full scalar invariance was not achieved, the comparison in mean differences of the constructs was restricted to the item level rather than the latent means.

**TABLE 5:** Fit indices and model comparisons of measurement invariance testing with grade levels as the grouping variable.

**Model**	**LL**	**SCF**	**Npar**	**RMSEA**	**CFI**	**SRMR**	Δ**RMSEA**	Δ**CFI**	Δ**SRMR**	Model comparisons^a^ ΔSB-χ^2^ (Δ*df*)
Self-efficacy										
Configural invariance	−5187.1	1.02	128	0.075	0.959	0.040				
Metric invariance	−5196.7	1.08	101	0.070	0.957	0.054	−0.005	−0.002	0.014	24.0 (27)
Time constraints										
Configural invariance	−4849.6	1.09	76	0.047	0.984	0.034				
Metric invariance	−4851.9	1.12	61	0.022	0.995	0.042	−0.025	0.011	0.008	4.6 (15)
CAS: General and inquiry										
Configural invariance	−5989.71	1.31	148	0.043	0.973	0.052				
Metric invariance	−6001.68	1.33	121	0.036	0.977	0.063	−0.007	0.004	0.011	20.5 (27)

LL, log-likelihood value; SCF, scaling correction factor; Npar, number of parameters; RMSEA, root mean square error of approximation; CFI, comparative fit index; SRMR, standardized root mean square residual; SB-χ^2^, Satorra–Bentler corrected chi-square statistic; *df*, degrees of freedom. ^*a*^ The difference test for model comparisons is based on Satorra–Bentler chi-square difference test, which produced corrected Δχ^2^ statistics when MLR is used as the maximum-likelihood estimator. ^*^p < 0.05, ^∗∗^*p* < 0.01, ^∗∗∗^*p* < 0.001.

#### Structural Relations

**FIGURE 3:**
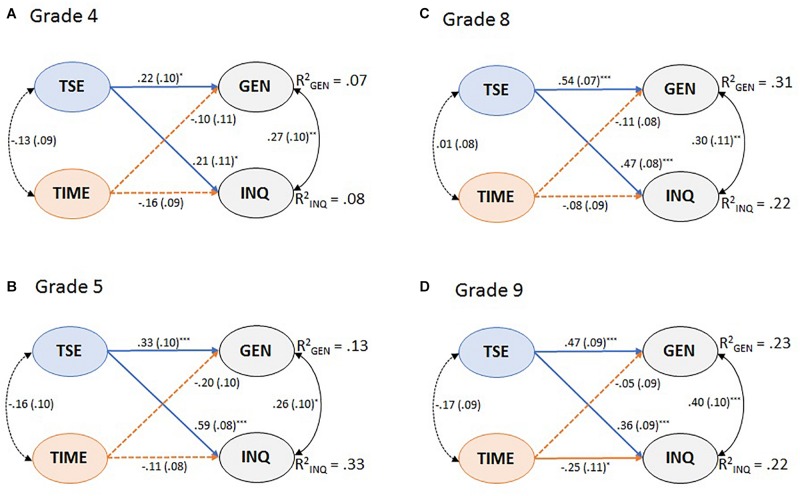
Multigroup structural equation model representing the relations among the latent variables across **(A)** Grade 4, **(B)** Grade 5, **(C)** Grade 8, and **(D)** Grade 9. Latent variables: TSE, teacher self-efficacy in science teaching; TIME, teachers’ perception of time constraints; GEN, general CAS; INQ, inquiry-based CAS. ^*^p < 0.05, ^∗∗^*p* < 0.01, ^∗∗∗^*p* < 0.001.

To examine the relationship differences due to grade levels, we established a multi-group model that combined all latent constructs under the assumption of metric invariance (Figure 3). This showed an acceptable fit to the data, SB-χ^2^(1317) = 1677.7, *p* < 0.001, RMSEA = 0.039, CFI = 0.947, TLI = 0.944, SRMR = 0.062. The explained variance across grades ranged between 7 and 31% for general CAS and between 8 and 33% for inquiry-based CAS. This model revealed that teacher self-efficacy in science teaching was positively related to both general and inquiry-based CAS, whereas teachers’ perceptions of time constraints were not associated with general CAS. We also found negative relations between the perceived time constraints and inquiry-based CAS, although this latter relation was only significant in Grade 9. These relations remained after controlling for teachers’ gender, their years of teaching experience, and their educational level for every grade level.

Although the signs of the relationships were similar across grades, their strengths varied to some extent. The relationships between teacher self-efficacy and general CAS were the strongest in Grade 8 (β = 0.54, *SE* = 0.07, *p <* 0.001) whereas the relationships between teachers’ self-efficacy and inquiry-based CAS had the largest path coefficient in Grade 5 (β = 0.59, *SE* = 0.08, *p <* 0.001).

#### Structural Invariance Testing

To further test whether the structural coefficients varied significantly across grade levels, we constrained the relations between the latent variables to be equal across grades and compared the constrained models with the baseline model that freely estimated these relations. As shown in Table 6, the structural relations among the constructs were significantly different across grade levels, except for the relations between the perceived time constraints and general CAS. As structural invariance was not attained for all the relationships, this provided evidence for significant differences in the structural relations.

**TABLE 6:** Fit indices and model comparisons of structural invariance testing with grade levels as the grouping variable.

**Multi-group model (across grades)**	**LL**	**SCF**	**Npar**	**RMSEA**	**CFI**	**SRMR**	Δ**RMSEA**	Δ**CFI**	Δ**SRMR**	Model comparisons^a^ ΔSB-χ^2^ (Δ*df*)
Freely estimated	−15,958.8	1.16	303	0.040	0.946	0.062				
Constrained the relations TSE → GEN	−15,963.8	1.16	300	0.040	0.945	0.066	0.000	00.001	−0.004	10.0 (3)^*^
Constrained the relations TSE → INQ	−15,964.7	1.16	300	0.040	0.945	0.068	0.000	00.001	−0.006	11.8 (3)^*^
Constrained the relations TIME → GEN	−15,962.4	1.16	300	0.040	0.945	0.064	0.000	00.001	−0.002	7.2 (3)
Constrained the relations TIME → INQ	−15,963.5	1.16	300	0.040	0.945	0.065	0.000	00.001	−0.003	9.5 (3)^*^

TSE, teacher self-efficacy in science teaching; GEN, general CAS; INQ, inquiry-based CAS; TIME, teachers’ perception of time constraints; LL, log-likelihood value; SCF, scaling correction factor; Npar, number of parameters; RMSEA, root mean square error of approximation; CFI, comparative fit index; SRMR, standardized root mean square residual; SB-χ^2^, Satorra–Bentler corrected chi-square statistic; *df*, degrees of freedom. ^*a*^ The difference test for model comparisons is based on Satorra–Bentler chi-square test, which produced corrected Δχ^2^ statistics when MLR is used as the maximum-likelihood estimator. All models with constraints were compared against the freely estimated model. ^*^p < 0.05, ^∗∗^*p* < 0.01, ^∗∗∗^*p* < 0.001.

Similar to the overall *F* -test in an ANOVA, this structural invariance testing procedure, however, only provides information about the existence of significance difference, yet not about where exactly these differences lie. Hence, to examine in which grade levels the differences in relations were statistically significant, we compared their strengths relative to one another using Wald tests (Table 7). For the relations between teacher self-efficacy and general CAS, the findings showed a significant difference between Grades 4 and 8 as well as between Grades 5 and 8, whereas for the relationships between teachers’ self-efficacy and inquiry-based CAS, the difference occurred between Grades 4 and 5 as well as between Grades 4 and 8.

**TABLE 7:** Differences in relations across grades.

**Relations**	**Grade comparisons Wald χ^2^ (*df*)**					
	**4 vs. 5**	**4 vs. 8**	**4 vs. 9**	**5 vs. 8**	**5 vs. 9**	**8 vs. 9**
TSE → GEN	0.29 (1)	5.53 (1)^*^	3.33 (1)	4.05 (1)^*^	1.69 (1)	0.35 (1)
TSE → INQ	6.13 (1)^∗∗^	4.29 (1)^*^	2.35 (1)	0.13 (1)	0.99 (1)	0.27 (1)

TSE, teacher self-efficacy in science teaching; GEN, general CAS; INQ, inquiry-based CAS. ^*^p < 0.05, ^∗∗^*p* < 0.01, ^∗∗∗^*p* < 0.001.

Taken together, the results suggested that (a) teachers who perceived themselves as more competent in science teaching reported a more frequent implementation of both general and inquiry-based CAS, for the overall sample and the sample across grade levels, and (b) teachers who perceived stronger time constraints in their classrooms enacted inquiry-based CAS less frequently, for the overall sample and for the subsample in Grade 9.

## Discussion

The goal of this study was to explore the relations among teachers’ self-efficacy in science teaching, the perceived time constraints, and the implementation of CAS in their classrooms. Our investigation extends previous research in two ways: First, it develops a deeper conceptual understanding of CAS by presenting empirical evidence for the distinction between generic and specific aspects of CAS. Second, it provides insights into the important roles of teachers’ self-efficacy and perceived time constraints for the enactment of CAS with data from Grades 4, 5, 8, and 9. The cross-grade comparisons further contribute to elucidating the differences between primary and secondary science teachers.

### Exploring General and Inquiry-Based CAS

The findings from our study showed that a two-factor model of CAS, distinguishing between general and inquiry-based CAS, was preferred against a one-factor model of CAS. The low correlation between both aspects of CAS suggests that they are distinct but related science teaching practices. From a *theoretical* perspective, general and inquiry-based CAS share similar features, and they are both aimed at engaging students in cognitively challenging learning activities. While general CAS typically pertain to activities common for many disciplines, such as activating students’ prior knowledge and linking the content to students’ everyday experience (Klieme et al., 2009; Baumert et al., 2010), inquiry-based CAS are unique to science as they typically include activities that reflect cognitive processes used by scientists during scientific practices (Rönnebeck et al., 2016). Although general CAS are crucial for enhancing student learning, its implementation alone is not sufficient for quality science instruction and should be complemented with opportunities to construct knowledge and foster scientific habits of mind through exploration and investigation (Windschitl et al., 2012; McNew-Birren and van den Kieboom, 2017). As both generic and specific CAS complement each another, understanding the relations between them is crucial to capturing how and the extent to which teachers engage in such practices, as well as what types of knowledge should be emphasized in teacher training and education to support CAS implementation. From an *empirical* perspective, the distinction between general and specific aspects of CAS provides greater understanding of the extent to which their implementations can be related to other constructs. For example, in the current study, teachers’ frequent use of general and inquiry-based CAS could be explained by their self-efficacy or perceived time constraints. Given the theoretical and empirical considerations above, our findings provide further insights into the different types of practices that can maximize students’ cognitive engagement.

### The Role of Teacher Self-Efficacy and Perceived Time Constraints

Findings from the overall sample indicated that teachers’ sense of efficacy and perceived time constraints are instrumental for the enactment of CAS. In particular, the relationships between CAS and teacher self-efficacy were approximately four times stronger than the relationship between CAS and perceived time constraints. This study expanded previous knowledge about teacher self-efficacy by exploring its separate relations with general and inquiry-based CAS (β = 0.37 and β = 0.42). We found that the magnitude of these relationships was generally higher compared to previous studies (Holzberger et al., 2013, 2014; Künsting et al., 2016). This could be attributed to the measure of teacher self-efficacy focusing on specific tasks in science teaching and the measure of separate aspects of CAS enhancing the conceptual alignment among the constructs under investigation. As Bandura (2006) suggested, a greater alignment between the teaching practices presented in the self-efficacy scale with those presented in the frequency of occurrence scale could strengthen the link between self-beliefs and teaching practices, ultimately resulting in larger correlations.

Teachers who felt low self-confidence in teaching science reported less frequent use of general and inquiry-based CAS. This association may reflect teachers’ inadequate science knowledge and beliefs about CAS that hinder them from using such approaches and lead them to favor low-risk instructions such as lecture-driven lessons (Murphy et al., 2007). For instance, enacting inquiry-based science teaching requires teachers to have strong subject matter and pedagogical content knowledge as well as positive attitudes about the role of inquiry in order to guide students in their investigations (Crawford, 2007; Buczynski and Hansen, 2010; Chichekian and Shore, 2016). As these issues may affect teacher self-efficacy and the enactment of CAS, they should be addressed appropriately during teacher training and professional development (Crawford, 2007; Sandholtz and Ringstaff, 2014; Menon and Sadler, 2016). For example, pre- or in-service teachers could be given opportunities to experience success in strengthening their science content with CAS and to reflect on those experiences in order to make explicit connections with their own teaching. In other words, fostering mastery experiences – both cognitive and enactive mastery experience in the context of CAS – may strengthen teachers’ self-efficacy (e.g., Palmer, 2011; Menon and Sadler, 2016; Pfitzner-Eden, 2016) and, ultimately, their implementation of CAS in classrooms.

In addition to low self-efficacy, teachers who perceived time constraints as a strong obstacle in their classrooms reported a less frequent use of inquiry-based CAS. Nevertheless, no significant link was found between perceived time constraints and general CAS implementation. This result is of particular relevance as it could contribute to the recent policy discussion about allocating more instructional time in science (Blank, 2013; Banilower et al., 2018; Yeşil Dağlı, 2018), especially for engaging students in scientific inquiry. Recent comparative surveys on instructional time spent on science showed that Norwegian classrooms devoted considerably fewer hours compared to other countries (TIMSS 2015 Report; Martin et al., 2016a). Compared to international averages, teachers spent 29% less time on science teaching per year in Grades 4 and 5 and 47% less time in Grades 8 and 9 (Nilsen and Frøyland, 2016). In comparison with general CAS, engaging students in complex and authentic inquiry learning is time-consuming in nature, and lack of time has been a common area of concern for many teachers (Murphy et al., 2007; Smolleck and Mongan, 2011; Chichekian and Shore, 2016). Previous studies have demonstrated the effectiveness of inquiry activities as a basis for quality teaching to enhance science achievement (e.g., Furtak et al., 2012; Lazonder and Harmsen, 2016). Inquiry instruction has also been shown to have greater impacts on science learning for students with non-mainstream backgrounds compared to direct instruction (Estrella et al., 2018). If teachers are to enact inquiry approaches, it is imperative that they also be provided with adequate time to design and elaborate well-thought lessons to provide high-quality science teaching for all.

### Differences Across Grade Levels

Our findings demonstrated that, at the item level, primary teachers reported lower self-efficacy as well as a less frequent implementation of inquiry-based CAS, compared to secondary teachers; the opposite was true for general CAS (Supplementary Table S1). As presented in Table 1, secondary teachers tended to have higher educational qualifications and science specialization. Using the same data for teachers in Grade 9, Kaarstein et al. (2016) found that Norwegian teachers who took at least 60 credits in science courses, regardless their subject areas, showed better instructional quality than others. Poor teacher knowledge and science teaching experiences have also been linked to primary teachers’ low confidence in teaching science and reluctance to enact challenging teaching approaches (Murphy et al., 2007; Powell-Moman and Brown-Schild, 2011; Menon and Sadler, 2016). Lack of resources is another major challenge for inquiry-based pedagogy in primary schools (Murphy et al., 2007; Buczynski and Hansen, 2010; Chichekian and Shore, 2016). In our sample, Norwegian primary schools reported considerably lower access to sufficient equipment and materials for science activities than did secondary schools (Martin et al., 2016a; Nilsen and Frøyland, 2016), which might explain why primary teachers resorted to a more frequent use of general rather than inquiry-based CAS.

The links between teacher self-efficacy and general CAS were weaker for primary teachers compared to secondary teachers (Figure 3 and Table 7). Even though, as classroom teachers, primary teachers seem to have a better chance to build a supportive classroom climate that is conducive for the implementation of CAS compared to secondary teachers (Ryan et al., 2015), this opportunity did not seem to translate into stronger relations between teacher self-efficacy and general CAS in primary schools. Teachers’ self-efficacy in science teaching could be an indicator of their content-based knowledge, which seems to be more important in later grades (Goe, 2007; Nilsen et al., 2018). As the science content in secondary schools is increasingly more complex and specialized, this type of knowledge plays a stronger role in determining teacher instructional practices (Goe, 2007; Kind, 2009).

The strength of the relationships between teacher self-efficacy and inquiry varied across grades, especially between Grades 4 and 5 as well as between Grades 4 and 8 (Figure 3 and Table 7). These variations might relate to the particularly high magnitude of correlation in Grade 5. This finding seems unique to the Norwegian schools and could be attributed to the transition in the curriculum cluster that divides learning goals into Grades 2–4, 5–7, and 8–10 (Ministry of Education and Research, 2006). In the latest Norwegian curriculum reform in 2006, inquiry-based teaching has been emphasized within the core competencies of the Budding Researcher, which comprise increasingly complex inquiry activities that span from primary to secondary schools. For instance, the competencies for Grades 2–4 include to describe, illustrate, and converse about one’s own observations from experiments and in nature, whereas in Grades 5–7, they place more emphasis on using instruments and systematized data, evaluating the results, and presenting the data. As such, compared to Grade 4, the cluster of learning goals for students in Grade 5 is more advanced, and they require, to a certain extent, explicit inquiry instruction. Although teacher self-efficacy in science teaching relates to the implementation of inquiry-based CAS in both primary and secondary classrooms, this belief seems to be more critical for fifth-grade teachers. The levels of their confidence could indicate the professional knowledge they have for understanding the complex curricular goals and how to achieve them through inquiry activities using limited instructional time and resources in primary schools.

Even though the evidence of negative relationships between teachers’ perceptions of time constraints and inquiry-based CAS was found in the overall sample (Figure 2), these findings could not be generalized across grade levels as the significant relationships only existed for the science teachers in Grade 9 (Figure 3). Providing teachers with adequate time for conducting inquiry is essential regardless of grade levels; however, it seems to be particularly crucial for ninth-grade teachers in our data. This might be due to the increasing pressure teachers experience to prepare students for examination at the end of secondary school (Grade 10). Studies have shown that high-stakes testing presents a distinctive impact on teacher efforts to reform their practices toward inquiry-oriented teaching (Crawford, 2007; Chichekian and Shore, 2016). Even though prior research has demonstrated the significance role of inquiry approaches in promoting student achievement (e.g., Blanchard et al., 2010; Estrella et al., 2018; Teig et al., 2018), the enactment of authentic inquiry practice remains a challenge for many teachers in light of accountability pressures.

### Limitations and Future Directions

As this study presented a secondary analysis of TIMSS data, several limitations need to be considered: First, although we applied robust methods for analyzing the relations among latent instead of manifest variables and validated the findings across four grade levels, we cannot draw inferences about cause-and-effect relationships given the cross-sectional nature of the data. By taking a longitudinal perspective, future studies could establish whether these associations are causal and further investigate mediating variables that might affect the relationships demonstrated in this study. Second, the data were based on teachers’ self-reports rather than student reports or classroom observations. Hence, our conclusions are established from the teacher perspective on the constructs under investigation. Even though TIMSS assessed students’ perceptions of science teaching, these perceptions were neither completely aligned with those obtained from teachers nor with the teacher self-efficacy measure. Hence, within these limits of the TIMSS questionnaire design and selection of measures, the choice for teacher perceptions – as the perceptions of science teaching that were best-aligned with the self-efficacy measure – was the most justifiable. Nevertheless, we believe that adding further sources of information about the actual implementation of CAS in science classrooms, such as through video observations and classroom discourse, could enhance the robustness of our findings. Finally, although it is not necessarily a limitation of the present study, we acknowledge that the effectiveness of inquiry instruction in improving student achievement has been challenged. For example, a recent study by Jerrim et al. (2019) demonstrated that the high level of inquiry activities was not associated with science performance. Mixed findings in the literatures could relate to the ways both constructs were operationalized, measured, and analyzed. Even though the current study did not investigate the inquiry–achievement relationships, future studies could examine whether teacher beliefs play an important role in moderating the relationships.

## Conclusion

This research provides important insights into teachers’ beliefs about themselves and the perceived time constraints in explaining the opportunities for students to engage in cognitively challenging learning activities. It enhances our understanding about challenging instruction by providing empirical evidence on the distinction between the general and specific aspects of CAS. The analyses conducted in the current study covered beyond the descriptive statistics and bivariate relations among teacher constructs, as currently presented in TIMSS’ international and national reports (e.g., Bergem et al., 2016b; Martin et al., 2016a). For instance, it specifically evaluated the invariance of teacher constructs, examined multivariate relations for testing theory-driven models, and assessed whether these relations varied across subgroups within a country sample in order to enhance the robustness of the TIMSS reports. In particular, findings from the overall sample revealed positive links between teachers’ self-efficacy in science teaching and the implementation of general and inquiry-based CAS as well as negative relationships between teachers’ perception of time constraints and their frequent use of inquiry-based CAS. These findings were robust across Grades 4, 5, 8, and 9, except for the relations between perceived time constraint and inquiry-based CAS, which was only significant for the ninth-grade teachers. This study also adds to the existing research by comparing the relations between teachers’ self-efficacy and their enactment of CAS in primary and secondary education, as research in this area is relatively scarce. Our study contributes to the current discussion on promoting the importance of teachers’ beliefs about their teaching competences to foster the enactment of CAS in science classrooms. In addition, these results can stimulate a productive conversation between policymakers and other stakeholders about the possibility of allocating more time for CAS that aimed for implementing inquiry-based instruction. This dialogue must advance as reforms in science education continue to embrace inquiry-based pedagogy as the core of science curricula.

## Ethics Statement

This research is exempt from ethics approval. All data from this study are publicly available on https://timssandpirls.bc.edu/timss2015/international-database/ under the International Association for the Evaluation of Educational Achievement (IEA), which is responsible for conducting high-quality, large-scale comparative studies of education across the globe.

## Author Contributions

NT was the lead author in conceptualizing the research, conducting data analysis, and writing the manuscript. RS made substantial contribution in guiding the statistical analyses. RS and TN contributed significantly to all steps of the research as well as critically reviewed and revised the manuscript.

## Conflict of Interest Statement

The authors declare that the research was conducted in the absence of any commercial or financial relationships that could be construed as a potential conflict of interest.

## Footnotes

1 https://timssandpirls.bc.edu/timss2015/questionnaires/index.html

2 https://timssandpirls.bc.edu/timss2015/international-database/
