# Supplementary material for: I Know I Can, but Do I Have the Time? The Role of Teachers’ Self-Efficacy and Perceived Time Constraints in Implementing Cognitive-Activation Strategies in Science
Source: Front Psychol. 2019 Aug 2;10:1697. doi: 10.3389/fpsyg.2019.01697 (PMC6687835; doi:10.3389/fpsyg.2019.01697)
Supplement: Supplementary file 1 [file Table_1.docx]

# Supplementary Material

**Contents**

| A) | The phases of inquiry-based CAS in our study (a simplified inquiry-based learning framework from Pedaste et al., 2015). | Figure S1 |
| --- | --- | --- |
| B) | Item information and descriptive statistics | Table S1 |
| C) | The scree plot for CAS-items with reference values of the Empirical Kaiser Criterion (EKC) method | Figure S2 |
| D) | Measurement model of (a) teacher self-efficacy in science teaching, (b) teacher self-efficacy in science teaching with correlated errors, (c) perceived time constraint, and (d) perceived time constraint with correlated errors | Figure S3 |
| E) | Model fit statistics for the measurement models of teachers’ self-efficacy and perceived time constraints | Table S2 |
|  |  |  |

1. **Figure S1.** The phases of inquiry-based CAS in our study (a simplified inquiry-based learning framework from Pedaste et al., 2015).

**
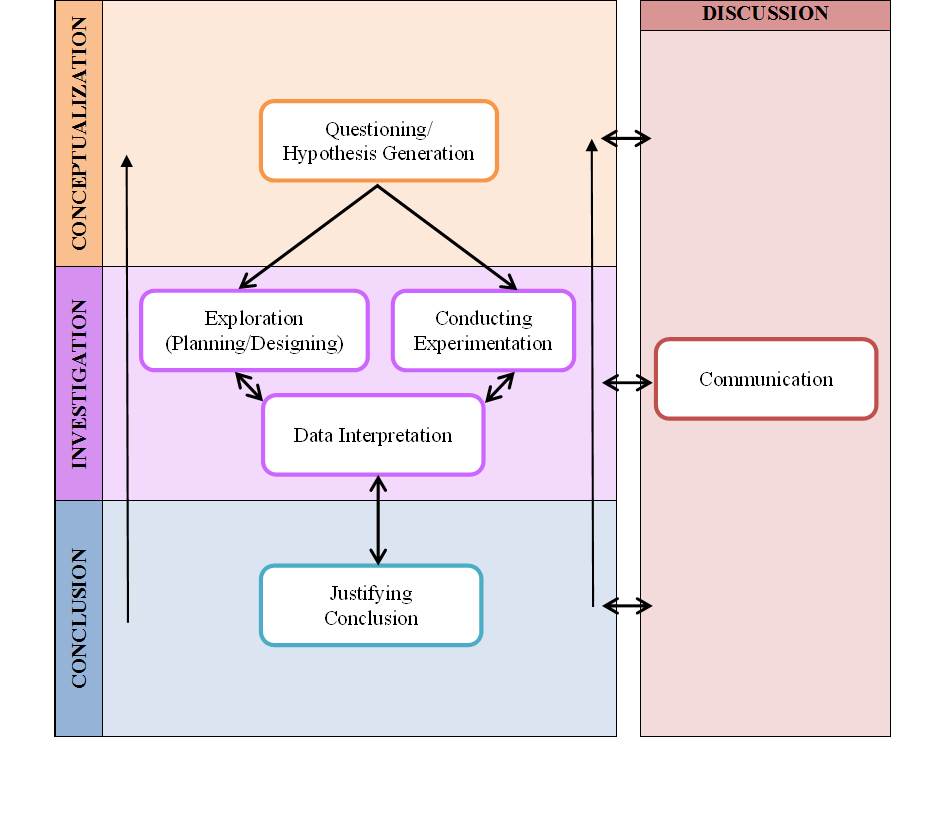
**

1. **Table S1.** Item information and descriptive statistics

| Item Label | Item Wording | Full sample  *N* = 804 | | Grade | | | | | | | |
| --- | --- | --- | --- | --- | --- | --- | --- | --- | --- | --- | --- |
|  |  |  |  | 4  *n* =193 | | 5  *n* =187 | | 8  *n* = 213 | | 9  *n* = 211 | |
|  |  | *M* | *SD* | *M* | *SD* | *M* | *SD* | *M* | *SD* | *M* | *SD* |
| *Teacher self-efficacy in science teaching (TSE)* | |  |  |  |  |  |  |  |  |  |  |
| Inspire | Inspiring students to learn science | 2.21 | 0.66 | 2.12 | 0.64 | 2.10 | 0.73 | 2.29 | 0.63 | 2.32 | 0.64 |
| Explain | Explaining science concepts or principles by doing science experiments | 1.81 | 0.79 | 1.61 | 0.75 | 1.61 | 0.84 | 2.03 | 0.76 | 1.91 | 0.74 |
| Tasks | Providing challenging tasks for the highest achieving students | 1.56 | 0.73 | 1.37 | 0.71 | 1.44 | 0.71 | 1.74 | 0.74 | 1.66 | 0.71 |
| Engage | Adapting my teaching to engage students’ interest | 1.82 | 0.68 | 1.76 | 0.66 | 1.80 | 0.70 | 1.86 | 0.71 | 1.86 | 0.66 |
| Value | Helping students appreciate the value of learning science | 1.99 | 0.66 | 1.95 | 0.65 | 1.93 | 0.70 | 2.02 | 0.64 | 2.03 | 0.65 |
| Assess | Assessing student comprehension of science | 1.88 | 0.66 | 1.71 | 0.63 | 1.73 | 0.69 | 1.98 | 0.65 | 2.04 | 0.61 |
| Unders | Improving the understanding of struggling students | 1.69 | 0.68 | 1.64 | 0.66 | 1.63 | 0.71 | 1.69 | 0.64 | 1.77 | 0.71 |
| Relvnt | Making science relevant to students | 1.94 | 0.64 | 1.92 | 0.61 | 1.91 | 0.69 | 1.96 | 0.65 | 1.98 | 0.61 |
| Think | Developing students’ higher-order thinking skills | 1.87 | 0.66 | 1.86 | 0.65 | 1.81 | 0.67 | 1.88 | 0.67 | 1.92 | 0.65 |
| Inquiry | Teaching science using inquiry methods | 1.58 | 0.72 | 1.48 | 0.71 | 1.53 | 0.76 | 1.65 | 0.71 | 1.63 | 0.72 |
| *Perceived time constraint (TIME)* | |  |  |  |  |  |  |  |  |  |  |
| Student | There are too many students in the classes | 1.85 | 1.03 | 1.74 | 1.06 | 1.64 | 1.08 | 2.03 | 0.98 | 1.85 | 1.03 |
| Materl | I have too much material to cover in class | 1.90 | 0.81 | 1.82 | 0.82 | 1.85 | 0.82 | 1.95 | 0.78 | 1.90 | 0.81 |
| Hour | I have too many teaching hours | 1.58 | 0.92 | 1.59 | 0.91 | 1.65 | 0.90 | 1.54 | 0.91 | 1.58 | 0.92 |
| Prep | I need more time to prepare for class | 2.04 | 0.86 | 2.15 | 0.87 | 2.09 | 0.87 | 2.02 | 0.82 | 2.04 | 0.86 |
| Assist | I need more time to assist individual students | 2.61 | 0.58 | 2.69 | 0.51 | 2.59 | 0.68 | 2.61 | 0.56 | 2.61 | 0.58 |
| Admin | I have too many administrative tasks | 1.97 | 0.93 | 2.07 | 0.85 | 1.93 | 0.95 | 1.97 | 0.98 | 1.97 | 0.93 |
| *General CAS (GEN)* | |  |  |  |  |  |  |  |  |  |  |
| Live | Relate the lesson to students’ daily lives | 2.11 | 0.78 | 2.14 | 0.76 | 2.04 | 0.79 | 2.10 | 0.80 | 2.15 | 0.75 |
| Chal | Ask students to complete challenging exercises that require them to go beyond the instruction | 1.36 | 0.66 | 1.37 | 0.70 | 1.34 | 0.70 | 1.38 | 0.62 | 1.34 | 0.65 |
| Disc | Encourage classroom discussions among students | 1.82 | 0.76 | 1.84 | 0.78 | 1.90 | 0.77 | 1.75 | 0.72 | 1.79 | 0.75 |
| Link | Link new content to students’ prior knowledge | 2.36 | 0.68 | 2.36 | 0.66 | 2.37 | 0.71 | 2.33 | 0.66 | 2.36 | 0.68 |
| Prob | Ask students to decide their own problem-solving procedures | 1.50 | 0.75 | 1.60 | 0.75 | 1.53 | 0.77 | 1.48 | 0.77 | 1.39 | 0.69 |
| Idea | Encourage students to express their ideas in class | 2.03 | 0.79 | 2.17 | 0.76 | 2.16 | 0.76 | 1.93 | 0.83 | 1.90 | 0.79 |
| *Inquiry-based CAS (INQ)* | |  |  |  |  |  |  |  |  |  |  |
| Expl | Design or plan experiments or investigations | 1.13 | 0.55 | .98 | 0.50 | 1.14 | 0.51 | 1.19 | 0.57 | 1.19 | 0.59 |
| Expr | Conduct experiments or investigations | 1.32 | 0.55 | 1.06 | 0.43 | 1.31 | 0.52 | 1.44 | 0.57 | 1.43 | 0.58 |
| Data | Interpret data from experiments or investigations | 1.08 | 0.48 | .94 | 0.44 | 1.12 | 0.49 | 1.10 | 0.47 | 1.16 | 0.48 |
| Com | Present data from experiments or investigations | 1.09 | 0.47 | .96 | 0.40 | 1.12 | 0.48 | 1.11 | 0.48 | 1.09 | 0.47 |
| Con | Use evidence from experiments or investigations to support conclusions | 1.16 | 0.57 | .98 | 0.55 | 1.09 | 0.57 | 1.25 | 0.57 | 1.26 | 0.56 |

1. **Figure S2.** The scree plot for CAS-items with reference values of the Empirical Kaiser Criterion (EKC) method **
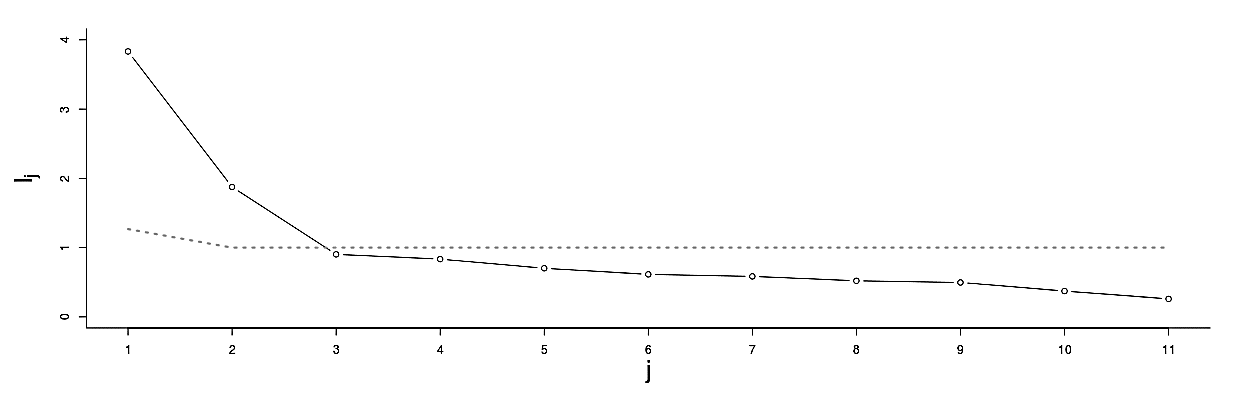
**

*Note.* I_j_ = eigenvalue number; j = observed eigenvalue (factor). The dotted line represent the reference values of the EKC method. According to the EKC, two factors should be selected as the third eigenvalue was the first one below its reference. Figure S3 was generated in <https://cemo.shinyapps.io/EKCapp/> based on the study from Braeken and van Assen (2017).

1. **Figure S3.** Measurement model of (a) teacher self-efficacy in science teaching, (b) teacher self-efficacy in science teaching with correlated errors, (c) perceived time constraint, and (d) perceived time constraint with correlated errors


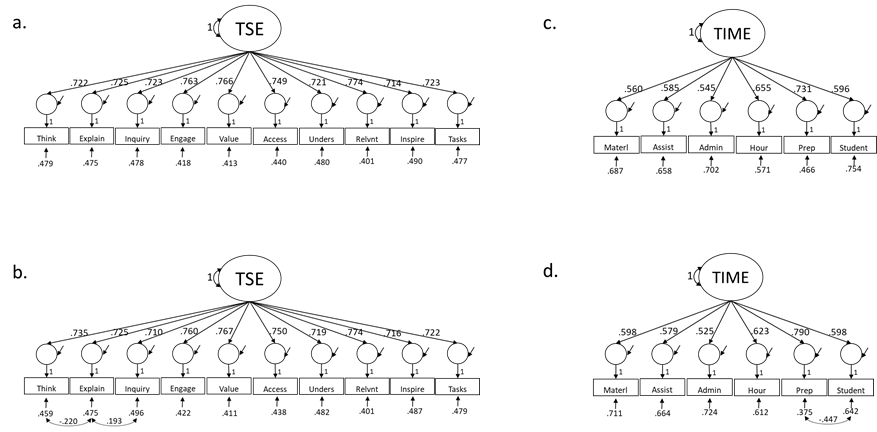


*Note.* Latent variables: TSE = teacher self-efficacy in science teaching; TIME = teachers’ perception of time constraint. Please refer to Table S2 for further details of the item labels and wordings as well as descriptive statistics of these measures.

1. **Table S2.** Model fit statistics for the measurement models of teachers’ self-efficacy and perceived time constraints

| Model | LL | SCF | Npar | RMSEA | CFI | SRMR | ΔRMSEA | ΔCFI | ΔSRMR | Model Comparisons^a^  ΔSB-χ^2^ (Δ*df*) |
| --- | --- | --- | --- | --- | --- | --- | --- | --- | --- | --- |
| *Teacher self-efficacy in science teaching* |  |  |  |  |  |  |  |  |  |  |
| Full sample |  |  |  |  |  |  |  |  |  |  |
| One-factor model | -5309.7 | 1.00 | 30 | 0.082 | 0.948 | 0.034 |  |  |  |  |
| One-factor model with residuals | -5285.8 | 1.01 | 32 | 0.074 | 0.960 | 0.031 | 0.008 | -0.012 | 0.003 | 47.7 (2)^***^ |
| Grade 4 |  |  |  |  |  |  |  |  |  |  |
| One-factor model | -1154.9 | 1.12 | 30 | 0.114 | 0.904 | 0.051 |  |  |  |  |
| One-factor model with residuals | -1142.4 | 1.14 | 32 | 0.103 | 0.926 | 0.047 | 0.011 | -0.022 | 0.004 | 24.9 (2)^***^ |
| Grade 5 |  |  |  |  |  |  |  |  |  |  |
| One-factor model | -1191.5 | 0.95 | 30 | 0.084 | 0.955 | 0.039 |  |  |  |  |
| One-factor model with residuals | -1179.5 | 0.96 | 32 | 0.062 | 0.977 | 0.033 | 0.022 | -0.022 | 0.006 | 24.1 (2)^***^ |
| Grade 8 |  |  |  |  |  |  |  |  |  |  |
| One-factor model | -1191.5 | 0.96 | 30 | 0.062 | 0.968 | 0.039 |  |  |  |  |
| One-factor model with residuals | -1179.4 | 0.98 | 32 | 0.060 | 0.971 | 0.038 | 0.002 | -0.003 | 0.001 | 5.9 (2), *p* = .09 |
| Grade 9 |  |  |  |  |  |  |  |  |  |  |
| One-factor model | -1420.9 | 1.00 | 30 | 0.076 | 0.950 | 0.042 |  |  |  |  |
| One-factor model with residuals | -1414.2 | 1.00 | 32 | 0.068 | 0.962 | 0.040 | -0.008 | -0.012 | 0.002 | 13.6 (2)^**^ |
| *Perceived time constraints* |  |  |  |  |  |  |  |  |  |  |
| Full sample |  |  |  |  |  |  |  |  |  |  |
| One-factor model | -4926.3 | 1.17 | 18 | 0.099 | 0.919 | 0.041 |  |  |  |  |
| One-factor model with residuals | -4899.7 | 1.14 | 19 | 0.054 | 0.979 | 0.026 | 0.045 | -0.060 | 0.015 | 53.2 (1)^***^ |
| Grade 4 |  |  |  |  |  |  |  |  |  |  |
| One-factor model | -1157.7 | 1.14 | 18 | 0.064 | 0.949 | 0.045 |  |  |  |  |
| One-factor model with residuals | -1154.1 | 1.14 | 19 | 0.033 | 0.988 | 0.037 | 0.031 | -0.039 | 0.008 | 7.1 (1)^**^ |
| Grade 5 |  |  |  |  |  |  |  |  |  |  |
| One-factor model | -1158.4 | 1.19 | 18 | 0.113 | 0.910 | 0.049 |  |  |  |  |
| One-factor model with residuals | -1151.6 | 1.17 | 19 | 0.065 | 0.974 | 0.036 | 0.048 | -0.064 | 0.013 | 13.5 (1)^***^ |
| Grade 8 |  |  |  |  |  |  |  |  |  |  |
| One-factor model | -1264.1 | 1.10 | 18 | 0.095 | 0.938 | 0.043 |  |  |  |  |
| One-factor model with residuals | -1255.5 | 1.07 | 19 | 0.022 | 0.997 | 0.029 | 0.073 | -0.059 | 0.014 | 17.1 (1)^***^ |
| Grade 9 |  |  |  |  |  |  |  |  |  |  |
| One-factor model | -1296.3 | 1.05 | 18 | 0.103 | 0.923 | 0.045 |  |  |  |  |
| One-factor model with residuals | -1288.3 | 1.08 | 19 | 0.059 | 0.977 | 0.035 | 0.044 | -0.054 | 0.010 | 15.9 (1)^***^ |

*Note*: LL = Log-likelihood value; SCF = scaling correction factor; Npar = number of parameters; RMSEA = Root Mean Square Error of Approximation; CFI = Comparative Fit Index; SRMR = Standardized Root Mean Square Residual; SB-χ^2^ = Satorra-Bentler corrected chi-square statistic; *df* = degrees of freedom.

^a^ The difference test for model comparisons is based on Satorra-Bentler chi-square test, which produced corrected Δχ^2^ statistics when MLR is used as the maximum likelihood estimator.

^*^*p* < .05, ^**^*p* < .01, ^***^*p* < .001

**References**

Braeken, J., & van Assen, M. A. L. M. (2017). An empirical kaiser criterion. *Psychological Methods, 22*(3), 450-466. doi:10.1037/met0000074

Pedaste, M., Mäeots, M., Siiman, L. A., de Jong, T., van Riesen, S. A. N., Kamp, E. T., . . . Tsourlidaki, E. (2015). Phases of inquiry-based learning: Definitions and the inquiry cycle. *Educational Research Review, 14*, 47-61.
